# Spatial altitude factors on the association with myopia: a narrative review

**DOI:** 10.3389/fmed.2026.1841970

**Published:** 2026-07-20

**Authors:** Jian Huang, Xiaoqiong Li, Shunming Liu, Li Qi, Lei Liu

**Affiliations:** 1 Department of Ophthalmology, The First Affiliated Hospital of Jinzhou Medical University, Jinzhou, China; 2 Department of Ophthalmology, Guangdong Eye Institute, Guangdong Provincial People's Hospital (Guangdong Academy of Medical Sciences), Southern Medical University, Guangzhou, China; 3 Department of Pediatric Eye Disease, Baotou, Chaoju Eye Hospital, Baotou, China

**Keywords:** altitude, environmental exposure, hypoxia, myopia, refractive development, sunlight

## Abstract

Myopia has emerged as one of the most pressing global public health challenges, characterized by rapidly increasing prevalence, earlier onset, and growing risk of vision-threatening complications. While genetic susceptibility contributes to refractive development, accumulating evidence indicates that environmental exposures play a dominant role in shaping ocular growth trajectories. Spatial altitude represents a unique ecological exposure integrating hypobaric hypoxia, enhanced solar radiation, distinctive climatic conditions, and lifestyle patterns. Epidemiological observations consistently demonstrate lower myopia prevalence among high-altitude populations compared with lowland regions, suggesting potential protective environmental influences. This review synthesizes current epidemiological findings and mechanistic hypotheses regarding altitude-associated differences in refractive status. We discuss potential pathways involving light-induced dopaminergic signaling, hypoxia-mediated molecular regulation, behavioral adaptations, circadian synchronization, and gene–environment interactions. We further highlight limitations of existing evidence and propose future research directions integrating environmental monitoring, longitudinal cohort studies, and multi-omics approaches. Understanding altitude-related influences on ocular growth may provide novel insights into environmental interventions for myopia prevention and contribute to strategies addressing the global myopia epidemic.

## 1 Introduction

Myopia is a complex ocular condition characterized by excessive axial elongation resulting in refractive error and blurred distance vision. Beyond refractive inconvenience, high myopia substantially increases the lifetime risk of retinal detachment, myopic maculopathy, glaucoma, and irreversible visual impairment (1, 2). Over recent decades, the prevalence of myopia has increased dramatically worldwide, particularly in East and Southeast Asia, where adolescent prevalence frequently exceeds 80% (3). Projections suggest that nearly half of the global population may become myopic by 2050 (4), underscoring the urgency of identifying modifiable risk factors.

The rapid rise in myopia prevalence within a single generation cannot be explained by genetic evolution alone (5–8). Instead, environmental transformation associated with urbanization, educational intensification, and lifestyle changes is increasingly recognized as the primary driver. Traditional investigations have focused on individual behaviors including near work (9, 10) and limited outdoor activity (11). Regarding the development and progression of childhood myopia, growing evidence suggests that digital device use itself may not be the primary risk factor (12). Rather, the broader lifestyle transition toward increased indoor activities associated with device use may contribute to adverse effects on ocular development (13). This environmental shift is considered an important driver of the projected global rise in myopia prevalence. However, emerging perspectives emphasize broader ecological determinants capable of influencing visual development at the population level.

In addition to behavioral factors such as near work and outdoor exposure, a growing body of research has highlighted the role of broader environmental exposures in refractive development (14). These include ambient light intensity (15), urbanization level including built environment characteristics (16), climatic conditions (17), air pollution (18), green space (19), and seasonal variations (20). Such environmental factors may influence ocular growth through multiple pathways, including modulation of retinal neurotransmitters, circadian rhythm regulation, visual contrast conditions, and behavioral patterns associated with daily activity. Increasing attention has therefore been directed toward understanding how large-scale ecological environments shape visual development at the population level. Within this context, geographic and environmental gradients provide valuable opportunities to investigate how natural environmental variation affects refractive outcomes.

Spatial altitude provides a natural environmental experiment for investigating the ecological determinants of refractive development. Compared with lowland environments, high-altitude regions are characterized by reduced atmospheric pressure, lower partial oxygen pressure, increased ultraviolet and short-wavelength radiation exposure, greater diurnal temperature variation, lower humidity, and altered spectral composition of ambient light. In addition, reduced atmospheric scattering at higher elevations enhances ground-level illuminance and visual contrast. These environmental differences coexist with distinctive sociocultural and behavioral patterns, including variations in occupational structure, outdoor activity duration, educational intensity, and urbanization level.

**Figure 1:**
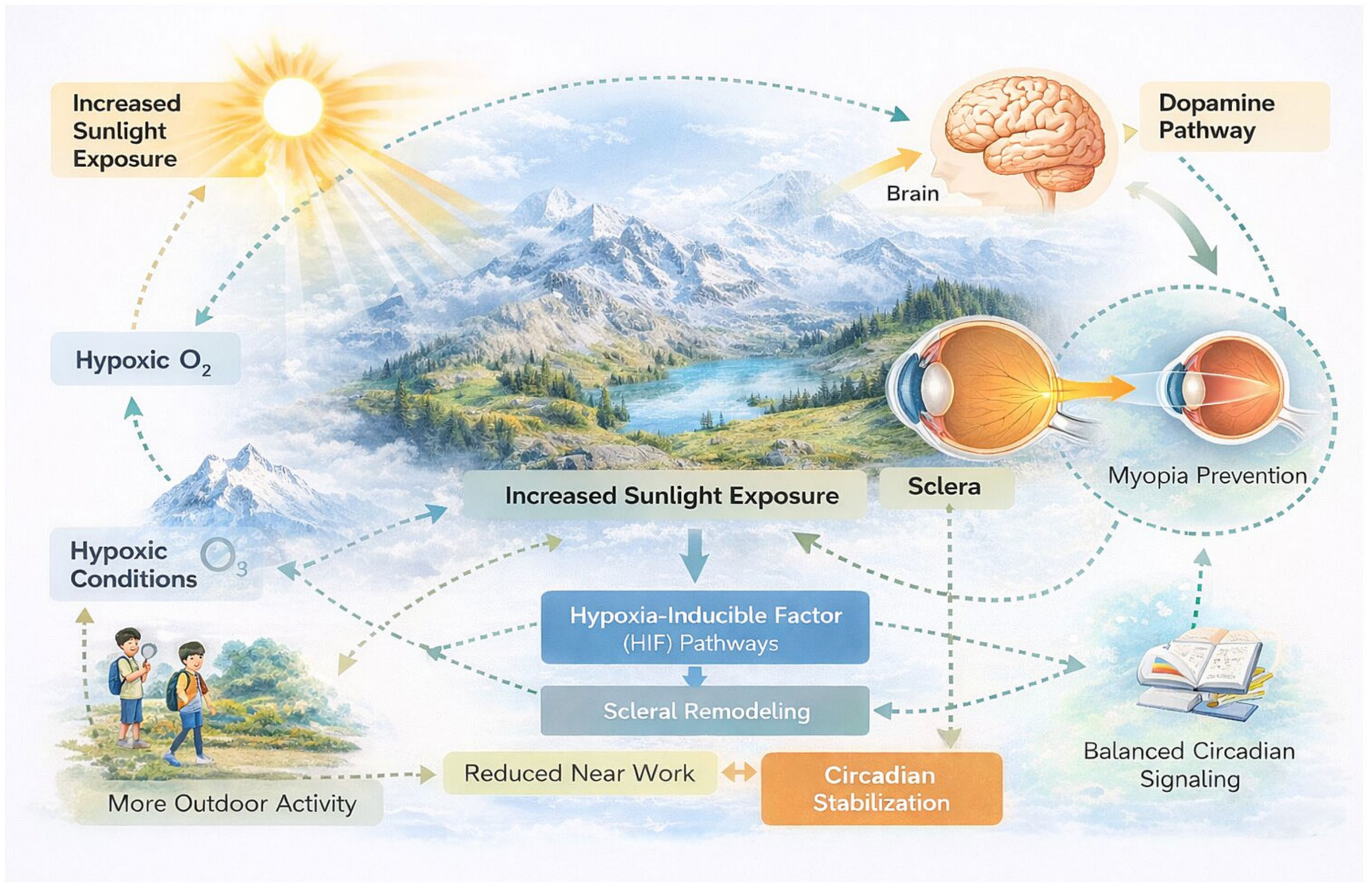
Graphic abstract of spatial altitude factors on the association with myopia.

Importantly, altitude-related exposures do not act in isolation but constitute an integrated environmental matrix that may influence ocular growth through both biological and behavioral pathways. Enhanced ambient illumination may modulate retinal dopamine release and circadian entrainment (15); hypobaric hypoxia may activate hypoxia-inducible signaling pathways affecting scleral extracellular matrix remodeling (21); and climatic as well as lifestyle characteristics may alter visual behavior and accommodative demand. Together, these multidimensional exposures create a unique ecological context in which environmental forces can be examined at a population level. Studying altitude-associated variation therefore offers a valuable opportunity to disentangle how large-scale environmental gradients shape refractive trajectories and to identify potentially protective factors that may be translated into preventive strategies for myopia control (Figure 1).

A structured literature search was conducted to identify relevant studies on altitude-related environmental exposures and myopia. We searched PubMed, Web of Science, and Embase databases from January 2000 to January 2026. The following keywords were used: (“altitude” OR “high altitude” OR “plateau”) AND (“myopia” OR “refractive error” OR “axial length”). Studies were included if they reported epidemiological or mechanistic associations between altitude-related environmental factors and refractive development in human or animal models. Review articles, original research, and relevant experimental studies were considered. No language restrictions were applied.

## 2 Epidemiological evidence of altitude-related differences in myopia

### 2.1 Prevalence patterns in high-altitude populations

At present, no universally accepted altitude threshold exists for myopia research. In environmental and physiological studies, elevations above 1,500 m are commonly considered moderate altitude, whereas elevations above 2,500 m are generally classified as high altitude. Given the heterogeneity of available studies, altitude should be interpreted as a continuous ecological gradient rather than a binary exposure variable. Population-based studies conducted across plateau regions including Tibetan, Andean, and Central Asian populations, consistently report lower prevalence of myopia among children compared with urban lowland populations. Generally, the prevalence of refractive errors in children and adolescents was significantly lower in Tibet (43.86%) than in Chongqing (53.80%). Furthermore, the Chongqing students had a longer AL than the group from Tibet (23.95 vs. 23.40 mm, respectively) (22). Ethnically, compared to the Han population, the Tibetan ethnicities were less likely to have myopia (23). Of note, the prevalence of myopia among Colombian adults aged 35–54 years was 14.4%, markedly lower than the 37.4% prevalence reported in a meta-analysis of European populations within the same age range (24). Notably, studies from Colombia may provide particularly informative ecological comparisons because Andean populations residing in cities such as Bogotá (~2,600 m) and Medellín (~1,400 m) can be compared with lower-altitude populations from coastal and plains regions within the same national context, thereby partially reducing confounding related to ethnicity and healthcare systems. Meta-analyses of Asian populations indicate that, within similar age groups and time periods, myopia prevalence tends to be lower in high-altitude countries such as Iran compared with low-altitude East Asian regions (25). Refractive distributions in these regions are frequently shifted toward emmetropia or mild hyperopia, suggesting delayed onset of myopic progression. While direct evidence is currently lacking, it is plausible that axial elongation may develop later during adolescence in plateau populations, implying that environmental exposures could modulate the timing of ocular growth rather than merely introducing refractive measurement bias. Many altitude comparisons involve rural plateau populations and urban lowland populations, introducing potential confounding from differences in education, lifestyle, healthcare access, and socioeconomic status.

It is important to acknowledge that substantial heterogeneity exists across these populations, including differences in ethnicity, socioeconomic status, urbanization level, educational intensity, and refractive measurement methods. Therefore, these findings should be interpreted as ecological comparisons rather than direct causal evidence of altitude effects. Residual confounding may partially or fully explain observed differences in myopia prevalence between high-altitude and low-altitude populations.

Where available, myopia was defined according to the conventional epidemiological criterion of spherical equivalent refraction (SER) ≤ −0.50 diopters. However, definitions varied across studies and may have contributed to heterogeneity in reported prevalence estimates.

### 2.2 Temporal changes and modernization effects

Recent longitudinal observations indicate a gradual increase in myopia prevalence even in plateau regions that were historically considered relatively protected from myopic development. In Colombia, for example, the prevalence of uncorrected refractive errors among eye care consultations reached 30.26%, increasing from 30.39% in 2015 to 35.14% in 2019 (26). Although these data do not exclusively represent myopia, the upward trend reflects a broader epidemiological transition in refractive disorders occurring alongside socioeconomic development. Similar patterns have been reported in several high-altitude regions undergoing rapid modernization, suggesting that environmental advantages associated with altitude may be progressively diminishing.

Educational expansion, widespread adoption of digital devices, and accelerated urbanization have substantially reshaped traditional lifestyles in plateau populations. Increased academic demands and prolonged screen exposure have led to longer durations of near work and reduced time spent outdoors, both of which are well-established environmental risk factors for myopia. Modern indoor environments also differ markedly from natural outdoor settings in terms of illumination intensity, spectral composition of light, and visual viewing distance, potentially altering retinal signaling pathways involved in ocular growth regulation. These lifestyle transitions may therefore counteract altitude-related environmental exposures that previously contributed to slower axial elongation.

In addition, the COVID-19 pandemic provided an unintended large-scale natural experiment for examining environmental influences on refractive development. Lockdown measures resulted in substantial reductions in outdoor activity and increased reliance on digital learning platforms. Multiple studies reported accelerated myopic progression during this period across diverse populations, including children living in high-altitude regions (27). The consistency of these observations across geographic settings highlights the dominant role of behavioral and environmental factors over geographic elevation alone.

Collectively, these findings reinforce the concept that altitude itself is unlikely to confer intrinsic biological protection against myopia. Rather, altitude-associated differences appear to operate through modifiable environmental pathways, including light exposure, lifestyle structure, and visual behavior. As modernization continues to alter environmental exposures worldwide, understanding how these factors interact with ecological contexts becomes increasingly important for developing effective population-level myopia prevention strategies.

## 3 Environmental characteristics of high-altitude regions

### 3.1 Enhanced solar radiation and ambient illumination

Reduced atmospheric thickness at high altitude increases ground-level solar irradiance and environmental brightness (28). Light intensity may increase by approximately 10–12% per 1,000 meters of elevation gain. High ambient illumination represents one of the most consistently identified protective factors against myopia (29, 30).

Bright light exposure has been associated with increased retinal dopamine signaling, which may play a role in regulating axial elongation (15). Dopamine (DA) influences retinal signaling cascades regulating scleral extracellular matrix remodeling, thereby stabilizing eye growth. Nevertheless, the biological mechanisms underlying light-induced increases in retinal dopamine signaling remain unclear. Given the complex and compensatory regulation of dopaminergic pathways in the retina, it is likely that multiple DA-associated proteins and signaling components participate in mediating the protective effects against myopia progression (15). Sustained exposure to intense natural illumination in plateau environments may therefore promote physiological emmetropization.

Furthermore, outdoor light provides a broad spectral composition distinct from artificial indoor lighting, potentially activating multiple photoreceptor pathways involved in ocular development.

### 3.2 Hypobaric hypoxia and physiological adaptation

Hypobaric hypoxia is a defining feature of high-altitude environments. Chronic exposure activates hypoxia-inducible factors (HIFs), which regulate angiogenesis, metabolism, and extracellular matrix turnover. These molecular processes are directly relevant to scleral remodeling and ocular biomechanics (21). But few studies have been concerned with scleral hypoxia and the underlying mechanism of how hypoxia may induce myopia is still unclear. Few evidence suggests oxygen tension may influence collagen synthesis and matrix metalloproteinase activity within ocular tissues. Mild chronic hypoxia may influence scleral remodeling processes; however, current evidence is limited and the direction and magnitude of its effect on axial elongation remain uncertain (31, 32). However, direct human evidence remains limited, highlighting an important area for future research.

### 3.3 Climatic and atmospheric influences

High-altitude climates are characterized by lower humidity, greater diurnal temperature variability, and enhanced visual contrast due to reduced atmospheric scattering. The increased visual contrast and clarity at high elevations may improve spatial resolution and contrast sensitivity, potentially reducing accommodative stress during distance viewing and thereby influencing ocular growth and refractive development (33, 34). Lower humidity may also affect tear film stability and ocular surface physiology, indirectly modulating visual quality and accommodative demands.

In addition to optical effects, extended daylight duration in high-altitude regions may reinforce circadian rhythm entrainment, which is known to regulate retinal neurotransmitter release, including dopamine, and ocular axial elongation (35). Seasonal and daily fluctuations in light exposure may further modulate melatonin signaling and retinal photoreceptor activity, creating additional temporal cues that influence ocular growth.

Climatic and atmospheric conditions may also interact with behavioral patterns. For instance, reduced atmospheric scattering and higher ambient illumination could encourage outdoor activity, amplifying protective effects against myopia. Moreover, variations in temperature and weather may influence habitual visual tasks, such as time spent reading or using digital devices, thereby indirectly shaping refractive trajectories.

## 4 Behavioral and lifestyle factors associated with altitude

### 4.1 Outdoor activity patterns

Outdoor activity duration is consistently associated with reduced myopia incidence. Plateau populations traditionally engage in outdoor labor, walking, and recreation, resulting in prolonged exposure to natural light. Evidence suggests a dose–response relationship, with approximately two hours of daily outdoor exposure providing significant protective effects (36). Objective quantification of environmental exposures, including outdoor activity duration, wearable light-sensor measurements, and ambient illuminance levels, remains largely unavailable in existing studies.

The interaction between altitude-related environmental brightness and outdoor behavior likely amplifies retinal dopamine signaling, reinforcing inhibitory control over axial elongation.

### 4.2 Educational structure and near work

Educational intensity is strongly associated with myopia risk (37, 38), primarily through prolonged near-work activities that increase accommodative demand and impose strain on the ciliary muscle. Historically, children and adolescents in plateau regions were exposed to lower academic pressure and correspondingly reduced near-work demands, which likely contributed to slower axial elongation and lower myopia prevalence in these populations. Educational intensity, reading duration, and near-work exposure may substantially confound altitude–myopia associations and should be carefully controlled in future studies.

However, rapid modernization has transformed educational environments. Extended school hours, increased homework load, and widespread adoption of digital learning platforms have substantially increased near-work duration, often in indoor settings with suboptimal illumination and limited visual breaks. Prolonged close-up tasks have been shown to elevate ocular accommodative lag and transient hyperopic defocus, both of which may stimulate compensatory axial elongation.

Additionally, increased screen exposure not only extends the duration of near work but also introduces higher temporal contrast and blue-light enrichment, which may differentially affect retinal dopamine signaling and circadian regulation (38). Modern educational practices often reduce opportunities for outdoor activity during school hours, further attenuating the protective effects of high ambient light exposure traditionally observed in plateau populations.

Together, these changes suggest that the historical environmental advantage conferred by lower educational intensity is progressively eroded. The interaction between intensified near work, reduced outdoor exposure, and environmental characteristics underscores the importance of considering both behavioral and ecological determinants when evaluating myopia risk in transitioning populations. Quantifying these combined effects is essential for designing population-level interventions that balance educational demands with ocular health.

## 5 Limitations of current evidence and research gaps

Existing studies are limited by cross-sectional designs and inconsistent refractive measurement protocols. Few investigations quantify environmental exposures objectively. Lack of wearable light sensors, oxygen monitoring, and standardized behavioral assessment limits causal inference.

In addition, substantial heterogeneity exists across study populations, including differences in ethnicity, socioeconomic status, urbanization, educational systems, and healthcare access. These factors may confound the observed associations between altitude and myopia. Most available studies are ecological in nature, limiting causal inference and increasing susceptibility to residual confounding. Publication bias toward positive associations may also influence the current evidence base. Importantly, heterogeneity in refractive assessment methods may also contribute to inconsistencies across altitude-related epidemiological studies. Studies using axial length as a structural marker of ocular elongation may more consistently reflect biological differences in myopia susceptibility (39), whereas studies relying primarily on spherical equivalent refraction may be influenced by corneal astigmatism and irregular corneal morphology (40, 41). High-altitude populations may exhibit increased prevalence of allergic ocular surface disease, keratoconus, or subclinical corneal ectasia, which could potentially lead to overestimation of myopia prevalence when refractive error alone is used as the primary outcome measure. Reverse causation cannot be excluded. Selective migration of highly educated and potentially more myopic individuals from plateau regions to urban lowland settings may contribute to observed population differences.

Taken together, the currently available evidence does not support a direct causal effect of altitude on myopia development. Rather, altitude may serve as a composite ecological exposure encompassing differences in ambient light, outdoor activity, educational intensity, urbanization, climatic conditions, and hypoxia-related physiology. Future longitudinal studies incorporating objective exposome measurements and multivariable adjustment will be required to disentangle the independent contribution of altitude-related environmental factors.

Future studies should integrate longitudinal cohort designs, environmental exposome measurements, and molecular biomarkers to clarify mechanisms linking altitude and refractive development. Moreover, this is a narrative review; therefore, no formal meta-analysis or risk-of-bias assessment was performed. Future systematic reviews with quantitative synthesis are warranted to validate and extend the present findings.

## 6 Public health implications and future perspectives

Altitude-associated observations provide valuable insights into scalable myopia prevention strategies. Increasing outdoor exposure, optimizing indoor illumination intensity, and designing educational environments aligned with circadian biology may replicate protective environmental effects observed in plateau populations.

Future studies should prioritize age-matched comparisons between high-altitude and low-altitude populations to minimize confounding arising from age-dependent differences in refractive development. High-altitude regions serve as natural laboratories for identifying environmental factors that can be translated into global public health interventions. Future studies should prioritize within-ethnicity comparisons or apply statistical adjustment for ethnic background to better isolate altitude-associated environmental effects. Future mechanistic studies may integrate systemic oxygen saturation measurements, hypoxia-inducible factor biomarkers, scleral extracellular matrix profiling, and experimental animal models to clarify the role of hypoxia in refractive development.

## 7 Conclusion

Altitude-associated environmental exposures influence ocular growth through multiple interconnected biological mechanisms. Increased light intensity at high elevations stimulates retinal dopamine release via amacrine cell activation, which modulates downstream pathways controlling scleral fibroblast activity and extracellular matrix remodeling, may contribute to signaling pathways involved in the regulation of axial elongation. Concurrently, hypobaric hypoxia activates HIF pathways, which regulate cellular metabolism, vascular adaptation, and interact with inflammatory mediators, potentially altering fibroblast proliferation, collagen cross-linking, and scleral biomechanical properties. Strong natural light–dark cycles may further stabilize circadian rhythms that govern retinal neurotransmitter release, providing an additional layer of environmental regulation of eye growth. These environmental influences operate in the context of genetic susceptibility, as populations adapted to hypoxia exhibit metabolic and vascular traits that may indirectly affect ocular development. Gene–environment interaction models suggest that altitude-related exposures modulate the expression of myopia susceptibility genes rather than acting independently, highlighting the synergistic interplay between ecological conditions and genetic predisposition in shaping refractive development.

Spatial altitude represents a multifactorial ecological exposure influencing myopia development through interconnected photic, hypoxic, behavioral, and physiological mechanisms. Enhanced illumination, lifestyle differences, and potential hypoxia-mediated biological pathways collectively contribute to reduced myopia susceptibility in plateau populations. However, modernization is progressively diminishing these protective influences. Interdisciplinary research integrating epidemiology, environmental science, and molecular ophthalmology is essential to elucidate altitude-related mechanisms and inform innovative strategies to combat the global myopia epidemic.
